# Urinary incontinence, core morphology and their impact on balance and fatigue in multiple sclerosis: an observational study

**DOI:** 10.3389/fneur.2026.1708066

**Published:** 2026-01-29

**Authors:** Cecilia Estrada-Barranco, Laura García-Ruano, Cristina-Belén Quilca-Esparza, Jacqueline-Maribel Tito-Torres, Marina Castel-Sánchez, Javier López-Ruiz, Marta de la Plaza-San Frutos

**Affiliations:** 1 Universidad Europea de Madrid, Faculty of Medicine, Health and Sports, Department of Physiotherapy, Villaviciosa de Odón, Madrid, Spain; 2 Fundación Esclerosis Múltiple de Madrid, Madrid, Spain

**Keywords:** balance, core muscle morphology, fatigue, multiple sclerosis, urinary incontinence

## Abstract

Introduction Multiple Sclerosis (MS) is a chronic condition affecting the central nervous system, often leading to urinary incontinence (UI), balance disturbances, and fatigue. This study examines the relationship between UI, core muscle morphology, balance, and fatigue in patients with MS (PwMS) to inform rehabilitation strategies. Methods A cross-sectional observational study was conducted with 27 PwMS (17 with UI and 10 without). Abdominal muscle thickness (transversus abdominis (TA), internal obliques, and external obliques) was assessed via ultrasound. UI-related Quality of Life was evaluated using questionnaires (ICIQ-SF and I-QOL), balance was assessed with the Trunk Impairment Scale (TIS) and Berg Balance Scale (BBS), and fatigue was measured using the Modified Fatigue Impact Scale (MFIS). Results Significant correlations were observed between UI, TA thickness during contraction and balance with the TIS demonstrating greater sensitivity than the BBS. PwMS with UI exhibited reduced TA thickness and poorer scores in balance and fatigue, particularly in the cognitive subscale of the MFIS. Logistic regression revealed that the severity of UI predicts functional balance, with an overall model accuracy of 70.8%. Conclusions Core dysfunction may link UI, balance and fatigue in PwMS. Strengthening the TA and pelvic floor muscles should be a rehabilitation priority to improve UI, postural stability, and daily function.

## Introduction

Multiple Sclerosis (MS) is a chronic, demyelinating, autoimmune, and inflammatory disease affecting the central nervous system (1). It is one of the leading causes of non-traumatic neurological disability in young adults aged 20 to 40 years. With a global prevalence exceeding 2.8 million individuals (2), its multifactorial aetiology involves genetic, immunological, and infectious factors (3). MS symptoms are diverse, affecting physical, cognitive, and emotional domains, significantly interfering with daily activities. Among these symptoms, fatigue, balance impairments, and urinary dysfunctions such as urgency, frequency, and urinary incontinence (UI) are particularly significant (4, 5). Urinary dysfunctions, including UI, affect approximately 60.4% of patients, with some cohorts reporting rates as high as 73.45% (6), making it one of the most frequent and debilitating complications.

Micturition control relies on a complex interaction between pelvic floor musculature and the passive stabilizing system, structures that also play a key role in postural control and respiration (7). The core, anatomically defined as a pressure cylindrical structure bounded by the diaphragm superiorly, the pelvic floor inferiorly, the deep abdominal muscles anteriorly, and the thoracolumbar fascia posteriorly, is essential for maintaining overall stability and functionality (8). In healthy individuals, a relationship has been described between pelvic floor dysfunction, including UI, and the activity of deep abdominal muscles (9, 10). The increase in intra-abdominal pressure through manoeuvres such as the Valsalva manoeuvre or coughing raises intra-abdominal pressure, which can lead to stress urinary incontinence, as well as gas or faecal incontinence, if the pelvic floor muscles are hypotonic and fail to provide retention. However, in the MS population, information regarding the connection between urinary dysfunction, core morphology, and its potential effects on balance and fatigue is limited (11, 12).

The evaluation of core morphology and its relationship with motor and urinary functions has been facilitated by the use of musculoskeletal ultrasound imaging (USI). This non-invasive, high-precision technique enables detailed analysis of the deep abdominopelvic musculature, overcoming limitations of other measurement tools (13, 14). In this context, USI emerges as a key tool to explore how morphological changes in the core may contribute to UI and its functional repercussions in patient with MS (PwMS).

This study focuses on analyzing the relationships between UI, core morphology, balance and fatigue in PwMS, utilizing advanced tools such as musculoskeletal ultrasound imaging and specific functional scales. The results could provide relevant insights for designing comprehensive rehabilitation strategies aimed not only at improving urinary dysfunction but also optimizing postural control and reducing fatigue, thereby significantly enhancing the quality of life in this population.

## Methods

An observational, cross-sectional study was conducted in individuals with MS, adhering to the Declaration of Helsinki (15) and the Personal Data Protection and Digital Rights Guarantee Act (Organic Law 3/2018) (16). The study followed the methodological guidelines of Strengthening the Reporting of Observational Studies in Epidemiology (STROBE) (17) to ensure research quality. Ethical approval was granted by the Ethics Committee of Hospital Clínico San Carlos in Madrid (code: 24/024-E). Participants were recruited through the Madrid Multiple Sclerosis Foundation (FEMM) and were thoroughly informed about the study.

**Figure 1:**
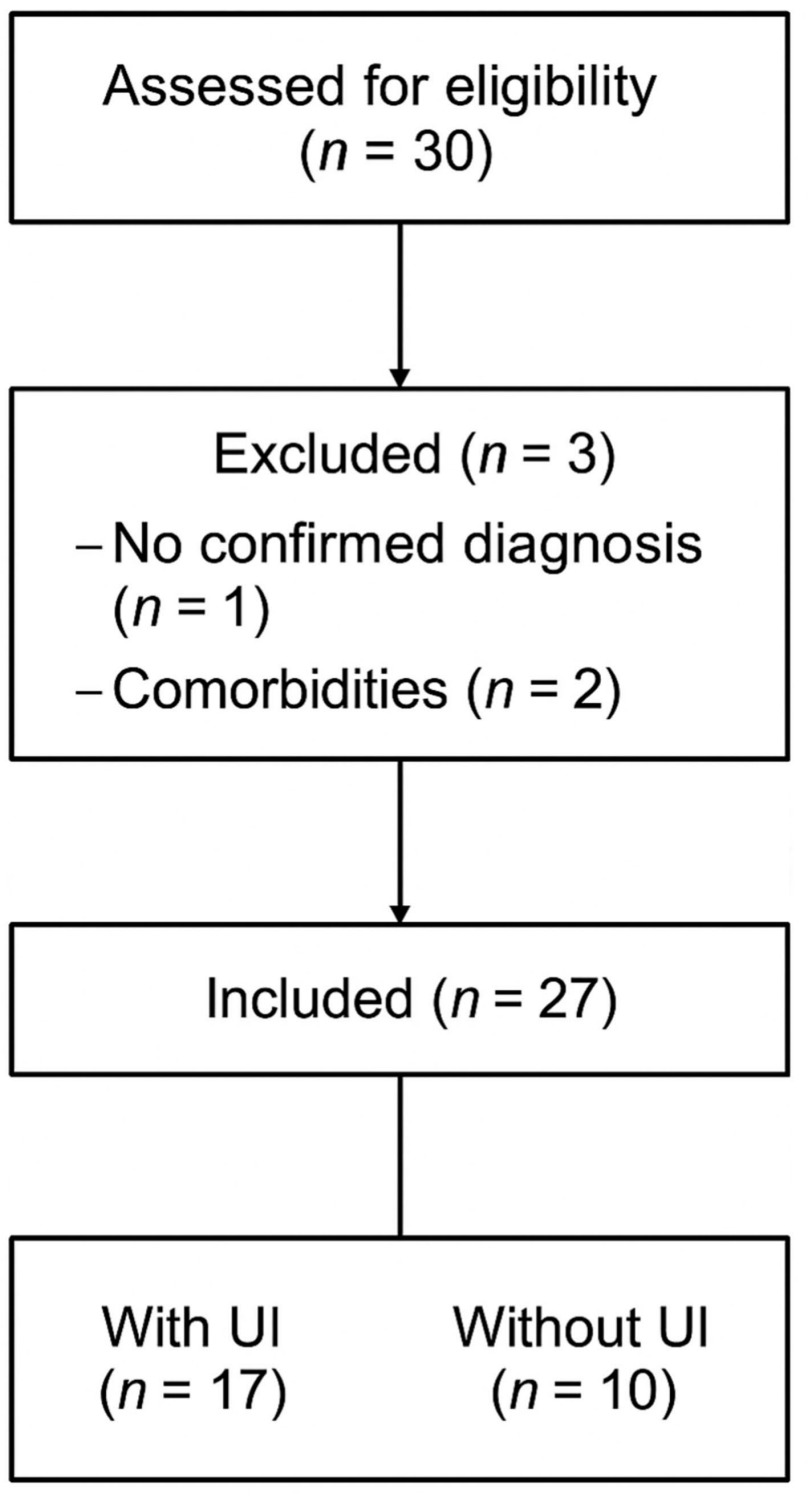
Flow diagram of participant recruitment and allocation. UI, Urinary incontinence.

Inclusion criteria included age between 18 and 70 years, a confirmed MS diagnosis based on 2017 McDonald’s criteria with disease progression of over 2 years, an EDSS score (18) between 2 and 7.5, stable medical treatment for at least 6 months, and no cognitive impairment (score ≥24 on the Mini-Mental Test) (19). Participants were required to present UI because of neurological impairment caused by MS. UI was identified using the International Consultation on Incontinence Questionnaire–Short Form (ICIQ-SF) with any total score greater than 0 considered indicative of UI, in accordance with the original instrument guidance. Exclusion criteria were other neurological or musculoskeletal conditions, factors that could interfere with the study, UI preceding the MS diagnosis, or steroid treatment within the last 6 months. Participant recruitment and allocation are illustrated in Figure 1.

Variables included age, weight, and height as independent variables. Dependent variables included UI-related quality of life, assessed using the Incontinence Quality of Life Questionnaire (I-QOL) and ICIQ-SF. The I-QOL is a self-reported, 22-item questionnaire measuring the impact of incontinence on a 0–100 scale, with higher scores indicating better quality of life (20). The ICIQ-SF assesses the severity of incontinence through three symptom-related items (frequency, amount of leakage and overall impact) and an additional question identifying situations of urine leakage (e.g., coughing or sneezing, physical activity, sudden urgency, or no obvious reason). Higher scores indicate greater symptom severity (21).

Balance was evaluated using the Trunk Impairment Scale (TIS) and the Berg Balance Scale (BBS). The TIS has a maximum score of 23 and assesses seated trunk control and segmental stability making it particularly suitable for detecting postural impairments in functional sitting positions. It comprises three subscales: static balance (TIS-ST), dynamic balance (TIS-DYN), and coordination (TIS-COOR) with higher scores indicating better postural stability (22). The BBS consists of 14 items with a total score of 56, evaluating functional balance in a standing position, with higher scores reflecting better balance (23). The use of both scales enabled the capture of complementary aspects of postural control: segmental trunk performance and overall standing balance.

Fatigue was measured using the Modified Fatigue Impact Scale (MFIS), a 21-item scale providing total and subscale scores across physical, cognitive, and psychosocial domains, where higher values indicate greater fatigue impact (24).

Baseline descriptive data were recorded to classify participants into groups with and without UI. Questionnaires, ultrasound measurements, and balance and fatigue tests were all completed in a single 40-min session during routine treatment visits at FEMM.

**Figure 2:**
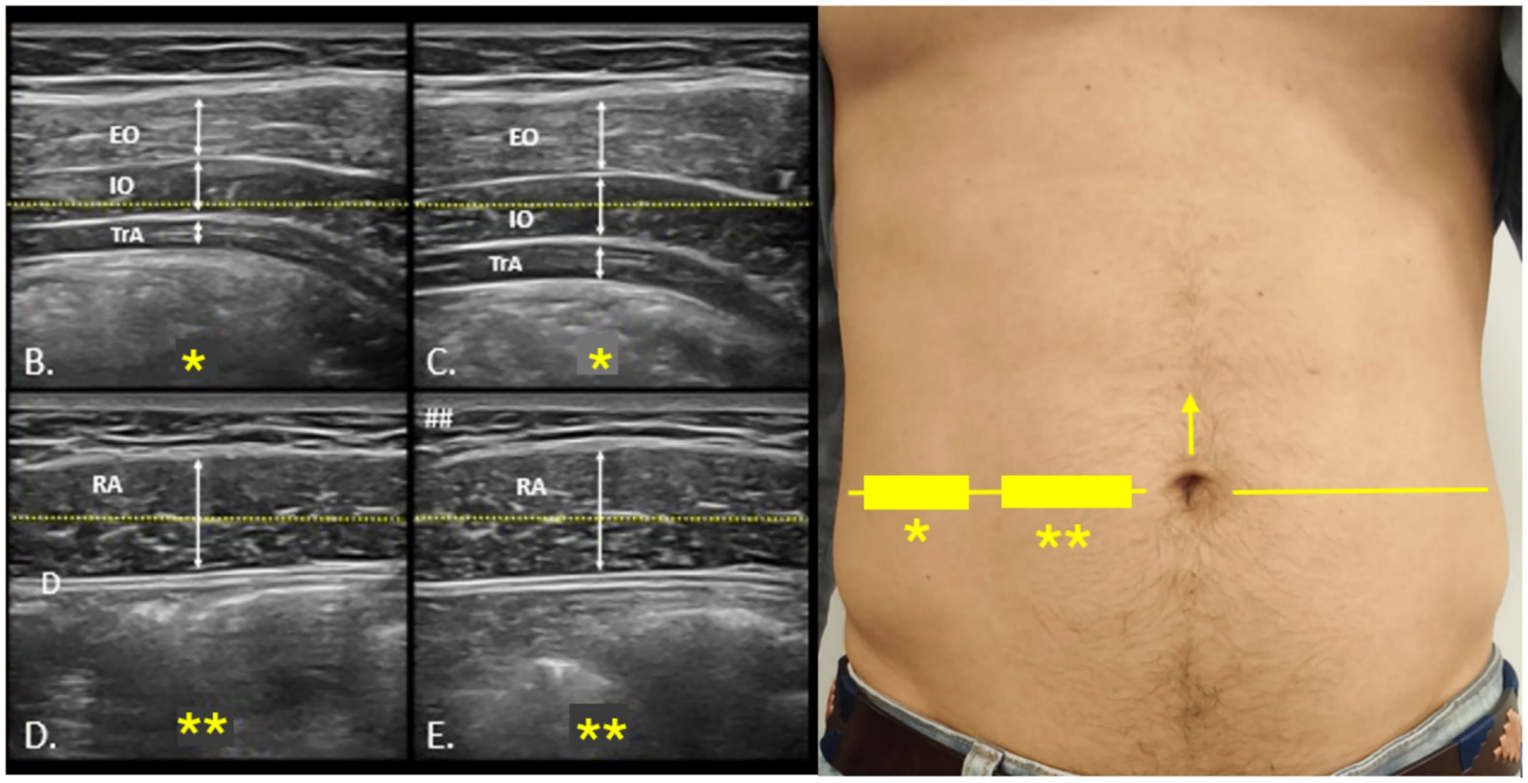
Ultrasound protocol for the evaluation of the anterolateral abdominal wall. Probe positioning for ultrasound assessment of the anterolateral abdominal wall **(A)**. The umbilical line, marked with a yellow arrow (→), is used as the reference for locating the lateral abdominal muscles and rectus abdominis, indicated on the skin with rectangles (▭), stars (*), and a horizontal yellow line (—). **(B,C)** show rest and contraction images of the lateral muscles, while **(D,E)** illustrate the same conditions for the rectus abdominis. EO, external oblique; IO, internal oblique; TrA, transversus abdominis; RA, rectus abdominis.

Core muscle morphology was assessed using a portable VSCAN® ultrasound device (GE Healthcare). Participants were positioned supine with comfortable hip and knee flexion. Generous gel was applied to minimize transducer pressure, and a high-frequency linear transducer was oriented transversely at the umbilical zone to visualize external oblique (EO), internal oblique (IO), and transversus abdominis (TA). Images were acquired at relaxed end-expiration when feasible, applying minimal probe pressure. Measurements included the thickness of TA, IO, and EO at rest and during contraction (25). All scans were performed by a single trained examiner following Rehabilitative Ultrasound Imaging (RUSI) standards to minimize measurement bias. For each condition, three measurements were obtained and averaged to ensure accuracy (26–29) (see Figure 2).

### Statistical analysis

For the statistical analysis, a comparison of the studied variables was conducted between PwMS who presented UI and those who did not. As the data did not follow a normal distribution, the Mann–Whitney U test was used. Spearman’s correlation was applied to evaluate the relationships between UI, core morphology, balance, and fatigue. Following Hopkins et al. (30), the following levels of correlation were established: trivial (*r* < 0.1), low (*r* ≥ 0.1 < 0.3), moderate (*r* ≥ 0.3 < 0.5), high (*r* ≥ 0.5 < 0.7), very high (*r* ≥ 0.7 < 0.9), and nearly perfect (*r* ≥ 0.9). A statistical significance level of *p* < 0.05 was considered.

To analyze the relationship between the severity of UI and postural balance, a binary logistic regression was performed. Functional balance, assessed with the BBS, was categorized as “low functional balance” (scores ≤45) or “good functional balance” (scores >45) (31). The predictor variable was the score obtained on the ICIQ-SF. The enter method was used, with entry and removal criteria set at 0.05 and 0.10, respectively. The model’s ability to correctly classify cases was evaluated using the classification table, the *χ*^2^ statistic for model coefficients, and pseudo-*R*^2^ values (32).

To ensure adequate precision in the results, the sample size was calculated using GRANMO© software. A sample of 29 participants was required to achieve a significance level of 0.05 and a statistical power greater than 0.8, assuming a correlation coefficient of 0.5.

## Results

A total of 27 were included in the study. Data were collected on age, sex, weight, height, and type of MS. Seventeen reported having stress incontinence and 10 did not have incontinence. The sociodemographic characteristics are shown in Table 1.

**Table 1:** Demographic and clinical characteristics by urinary incontinence status.

Variable	No incontinence (*n* = 10)	Incontinence (*n* = 17)	*p*-value
	x̅ ± *SD*	x̅ ± *SD*	
Age (years)	50.70 ± 8.99	45.94 ± 12.03	0.280
Weight (kg)	73.95 ± 9.30	70.24 ± 15.46	0.513
Height (cm)	173.20 ± 8.04	168.71 ± 7.89	0.174
Sex (M/F)	4/6	6/11	1.000
MS Type			0.568
Relapsing–remitting	8	12	
Secondary progressive	0	2	
Primary progressive	2	3	

Continuous variables are expressed as mean ± standard deviation. Categorical variables are expressed as absolute frequencies. *p*-values correspond to Mann–Whitney *U* test (continuous variables), Fisher’s exact test (Sex), and Fisher–Freeman–Halton exact test (MS Type). UI, urinary incontinence; MS, Multiple Sclerosis; M, male; F, female; x̅, Mean; SD, standard deviation.

The relationship between the two UI quality-of-life questionnaires (ICIQ-SF and I-QOL) and the balance and fatigue scales was analyzed. The ICIQ-SF demonstrated a very high correlation with the TIS-ST score and a high correlation with the global TIS score and TIS-COOR. A moderate correlation was found with TIS-DYN and the BBS. The I-QOL showed a high correlation with the TIS and TIS-ST, and a moderate correlation with TIS-DYN; however, no significant correlation was observed with the BBS or TIS-COOR.

Regarding fatigue, the ICIQ-SF demonstrated a high correlation with overall fatigue as well as with the cognitive subscale of the MFIS, and a moderate correlation with the physical subscale of the MFIS. The I-QOL showed a moderate correlation with the MFIS and its physical subscale, and a high correlation with the cognitive subscale of the MFIS.

When analyzing correlations by subgroups based on the presence or absence of UI, the TIS, TIS-ST, and TIS-DYN showed high to very high correlations with the ICIQ-SF in the group of patients with incontinence. No significant correlations were found in the continent group. These results are presented in Table 2.

**Table 2:** Relationship between incontinence-related quality of life, balance, and fatigue.

Group	Measure	TIS-ST *r* (*p*)	TIS-DYN *r* (*p*)	TIS-COOR *r* (*p*)	BBS *r* (*p*)	MFIS-total *r* (*p*)	MFIS-physical *r* (*p*)	MFIS-cognitive *r* (*p*)	MFIS-psychosocial *r* (*p*)
TOTAL	ICIQ-SF	**−0.63 (*p* < 0.001)**	**−0.73 (*p* < 0.001)**	**−0.49 (*p* = 0.01)**	**−0.54 (*p* = 0.01)**	**−0.48 (*p* = 0.02)**	**0.52 (*p* = 0.01)**	**0.49 (*p* = 0.01)**	**0.51 (*p* = 0.01)**
TOTAL	I-QOL	**0.62 (*p* = 0.001)**	**0.64 (*p* < 0.001)**	**−0.47 (*p* = 0.02)**	0.39 (*p* = 0.63)	0.36 (*p* = 0.88)	**−0.45 (*p* = 0.03)**	**−0.31 (*p* = 0.01)**	**−0.57 (*p* = 0.003)**
PwMS with UI	ICIQ-SF	**−0.58 (*p* = 0.01)**	**−0.64 (*p* = 0.01)**	**−0.46 (*p* = 0.01)**	−0.45 (*p* = 0.07)	−0.21 (*p* = 0.42)	0.44 (*p* = 0.07)	**0.51 (*p* = 0.04)**	0.27 (*p* = 0.29)
PwMS with UI	I-QOL	0.44 (*p* = 0.07)	0.45 (*p* = 0.07)	0.31 (*p* = 0.22)	0.19 (*p* = 0.46)	0.18 (*p* = 0.49)	−0.45 (*p* = 0.07)	−0.33 (*p* = 0.19)	−0.42 (*p* = 0.10)
PwMS without UI	ICIQ-SF	−0.15 (*p* = 0.68)	0.00 (*p* = 1.00)	−0.13 (*p* = 0.71)	0.10 (*p* = 0.78)	−0.27 (*p* = 0.45)	0.32 (*p* = 0.36)	0.34 (*p* = 0.34)	0.25 (*p* = 0.48)
PwMS without UI	I-QOL	−0.20 (*p* = 0.57)	−0.12 (*p* = 0.75)	−0.40 (*p* = 0.25)	−0.04 (*p* = 0.92)	0.53 (*p* = 0.11)	0.49 (*p* = 0.15)	**−0.31 (*p* = 0.01)**	0.18 (*p* = 0.62)

TIS, Trunk Impairment Scale; TIS-ST: Trunk Impairment Scale – Static subscale; TIS-DYN, Trunk Impairment Scale – Dynamic subscale; TIS-COOR, Trunk Impairment Scale – Coordination subscale; BBS, Berg Balance Scale; MFIS, Modified Fatigue Impact Scale; MFIS-Total, Modified Fatigue Impact Scale—Total score; MFIS-Physical, Modified Fatigue Impact Scale—Physical subscale; MFIS-Cognitive, Modified Fatigue Impact Scale—Cognitive subscale; MFIS-Psychosocial, Modified Fatigue Impact Scale – Psychosocial subscale; ICIQ, International Consultation on Incontinence Questionnaire-short form; I-QOL, Incontinence Quality of Life Questionnaire; PwMS with UI, patient with Multiple sclerosis with Urinary Incontinence; PwMS without UI, patient with Multiple sclerosis without Urinary Incontinence; *p*, *p*-value. Bold values indicate statistical significance, which was set at *p* < 0.05.

The relationship between abdominal muscle thickness, measured via ultrasound, and UI-related quality of life was also analyzed. A high correlation was found between the thickness of the IO during contraction and the ICIQ-SF score, and a moderate correlation was observed with IO thickness at rest. A moderate correlation was also established between the ICIQ-SF score and the TA thickness at rest. However, no significant correlation was found with the TA thickness during contraction or with the EO thickness at rest or during contraction. The I-QOL showed a moderate correlation only with EO thickness at rest.

As shown in Table 3, when dividing participants into groups based on the presence or absence of incontinence, a very high correlation was found in the contraction of the TA measured via ultrasound. Specifically, a greater thickness of the TA during contraction was strongly correlated with fewer incontinence symptoms.

**Table 3:** Relationship between incontinence-related quality of life and abdominal muscle thickness.

Group	Measure	TA-REST *r* (*p*)	IO-REST *r* (*p*)	EO-REST *r* (*p*)	TA-CONT *r* (*p*)	IO-CONT *r* (*p*)	EO-CONT *r* (*p*)
TOTAL	ICIQ-SF	−0.380 (*p* = 0.06)	**−0.428 (*p* = 0.03)**	−0.226 (*p* = 0.27)	0.033 (*p* = 0.877)	**−0.504 (*p* = 0.01)**	−0.234 (*p* = 0.231)
TOTAL	I-QOL	0.283 (*p* = 0.17)	0.247 (*p* = 0.23)	0.348 (*p* = 0.08)	−0.043 (*p* = 0.83)	0.341 (*p* = 0.09)	0.258 (*p* = 0.21)
PwMS with UI	ICIQ-SF	−0.164 (*p* = 0.528)	−0.311 (*p* = 0.224)	0.012 (*p* = 0.964)	**−0.577 (*p* = 0.015)**	−0.049 (*p* = 0.850)	0.144 (*p* = 0.581)
PwMS with UI	I-QOL	−0.273 (*p* = 0.445)	−0.102 (*p* = 0.779)	−0.110 (*p* = 0.763)	−0.124 (*p* = 0.732)	−0.285 (*p* = 0.424)	−0.221 (*p* = 0.539)
PwMS without UI	ICIQ-SF	−0.015 (*p* = 0.953)	−0.071 (*p* = 0.785)	−0.169 (*p* = 0.517)	0.394 (*p* = 0.118)	−0.344 (*p* = 0.176)	−0.251 (*p* = 0.331)
PwMS without UI	I-QOL	−0.303 (*p* = 0.396)	−0.370 (*p* = 0.292)	−0.144 (*p* = 0.692)	−0.288 (*p* = 0.420)	−0.514 (*p* = 0.128)	−0.069 (*p* = 0.851)

TA-REST, Transversus abdominis at rest; IO-REST, Internal oblique at rest; EO-REST, External oblique at rest; TA-CONT, Transversus abdominis during contraction; IO-CONT, Internal oblique during contraction; EO-CONT, External oblique during contraction; ICIQ-SF, International Consultation on Incontinence Questionnaire-short form; I-QOL, Incontinence Quality of Life Questionnaire; PwMS with UI, patient with Multiple sclerosis with Urinary Incontinence; PwMS without UI, patient with Multiple Sclerosis without Urinary Incontinence; *p*, *p*-value. Bold values indicate statistical significance, which was set at *p* < 0.05.

Table 4 shows the results of the comparison of all variables conducted between the group of patients with incontinence and those without using the Mann–Whitney U test. The results indicate that PwMS who experience incontinence exhibit higher levels of fatigue, reflected in the overall MFIS score and the cognitive subscale. They also demonstrate poorer balance, as measured by the BBS, and reduced muscle thickness (in mm) in the TA at rest and the IO at rest.

**Table 4:** Comparison between results of PwMS with and without UI expressed as median (interquartile range).

Variable	Median (IQR)–PwMS without UI	Median (IQR)–PwMS with UI	Mann–Whitney U	*p*-value
MFIS-total	40 (13)	50 (25)	44.000	***p* = 0.039**
MFIS-physical	27 (9.25)	30 (9.5)	59.500	*p* = 0.199
MFIS-cognitive	11 (9.25)	18 (15)	37.500	***p* = 0.017**
MFIS-psychosocial	3.5 (3.25)	5 (4.5)	66.000	*p* = 0.335
TIS-total	14.5 (4)	12 (6)	58.000	*p* = 0.171
TIS-ST	4 (1)	3 (1.5)	53.000	*p* = 0.083
TIS-DYN	7 (1.25)	6 (3)	67.500	*p* = 0.366
TIS-COOR	4 (1.25)	3 (2)	61.000	*p* = 0.191
BBS	46 (8.5)	40 (10)	38.500	***p* = 0.019**
TA-REST	0.325 (0.17)	0.200 (0.14)	39.000	***p* = 0.021**
IO-REST	0.750 (0.43)	0.480 (0.20)	37.500	***p* = 0.017**
EO-REST	0.385 (0.53)	0.350 (0.30)	62.500	*p* = 0.258
TA-CONT	0.430 (0.24)	0.320 (0.32)	61.000	*p* = 0.228
IO-CONT	0.865 (0.70)	0.780 (0.34)	52.500	*p* = 0.103
EO-CONT	0.490 (0.50)	0.400 (0.38)	71.000	*p* = 0.482

MFIS, Modified Fatigue Impact Scale; MFIS-Total, Total score; MFIS-Physical, Physical subscale; MFIS-Cognitive, Cognitive subscale; MFIS-Psychosocial, Psychosocial subscale; TIS, Trunk Impairment Scale; TIS-ST, Static subscale; TIS-DYN, Dynamic subscale; TIS-COOR, Coordination subscale; BBS, Berg Balance Scale; TA-REST, Transversus abdominis at rest; IO-REST, Internal oblique at rest; EO-REST, External oblique at rest; TA-CONT, Transversus abdominis during contraction; IO-CONT, Internal oblique during contraction; thickness of muscles measured in mm; EO-CONT, External oblique during contraction; IQR, interquartile range; PwMS with UI, patient with Multiple sclerosis with Urinary Incontinence; PwMS without UI, patient with Multiple Sclerosis without Urinary Incontinence; *p*, p-value. Bold values indicate statistical significance, which was set at *p* < 0.05.

A binary logistic regression was performed to evaluate whether the score on the ICIQ-SF predicts functional balance. BBS was dichotomized, the 45-point threshold corresponds to the original and most widely used cut-off for identifying increased fall risk in the BBS (23, 33), although it is not specific to MS. As shown in Table 5, the model indicated that the ICIQ-SF had a marginally significant inverse relationship with the likelihood of achieving good functional balance (*B* = −0.183, *p* = 0.054, Exp(B) = 0.833). The overall predictive capacity of the model was 70.8% (*χ*^2^ = 4.574, *p* = 0.032), with pseudo-*R*^2^ values indicating a moderate contribution of the ICIQ-SF. These results suggest that the severity of UI may influence postural balance, although other factors should also be considered.

**Table 5:** Binary logistic regression to evaluate ICIQ-SF as a predictor of balance.

Variable	*B*	*SE*	Wald	*df*	*p*	Exp(B)	95% CI for Exp(B)
ICIQ-SF	−0.18	0.10	3.71	1	*p* = 0.054	0.83	[0.69, 1.01]
BBS	0.84	0.72	1.37	1	*p* = 0.241	2.32	—

ICIQ-SF, International Consultation on Incontinence Questionnaire–Short Form; BBS, Berg Balance Scale; B, regression coefficient; SE, standard error; Wald, Wald statistic; df, degrees of freedom; *p*, *p*-value; Exp(B), odds ratio; CI, confidence interval. Statistical significance was set at *p* < 0.05.

## Discussion

In this study, 27 PwMS were evaluated, 17 of whom presented with stress UI. The results indicate that UI-related quality of life, assessed using the ICIQ-SF and I-QOL questionnaires, was significantly correlated with balance (TIS, BBS) and fatigue (MFIS) scales. Specifically, the ICIQ-SF showed a high correlation with balance and fatigue scores in the incontinent group, suggesting that this condition may be associated with greater postural control impairments and higher fatigue levels. Additionally, the analysis of TA muscle thickness during contraction revealed a negative correlation with ICIQ scores in PwMS with UI, emphasizing the importance of this muscle in postural control and incontinence severity.

Pelvic floor strengthening, including the TA, has been shown to effectively improve UI (34, 35). Its thickness during contraction appears to be more closely related to UI severity than other core muscles (36). The findings of the present study reinforce the hypothesis that core weakness may serve as a link between UI, fatigue, and balance (37). Moreover, the lack of a significant relationship between UI and EO thickness highlights the importance of targeting specific muscles in intervention strategies (34–36).

Regarding balance, patients with UI scored lower on the BBS, consistent with previous studies, such as that by Soll et al. (12), which reported an increased risk of falls in this population. However, the relationship between UI and postural control was captured more precisely by the TIS, which showed a significant correlation with the ICIQ-SF, particularly in the static balance subscale (−0.725). This suggests that the TIS, with its focus on segmental trunk control in functional positions, is more sensitive in detecting postural impairments related to incontinence than the BBS (38, 39), which evaluates global balance. Furthermore, the significant correlation between the TIS and the ICIQ-SF was observed only in the incontinent group, suggesting that these patients face greater challenges in activities requiring postural stability, such as position changes or daily tasks (40).

In contrast, the correlations between the I-QOL and the balance and fatigue scales were lower compared to the ICIQ-SF. This may be attributed to the I-QOL measuring the impact of incontinence on quality of life, which is influenced by multiple factors such as lower limb functionality (41) and the ability to perform transfers (42), whereas the ICIQ-SF directly assesses incontinence severity (43). This finding underscores that the core’s impact is more directly reflected in symptom severity than in the overall perception of quality of life.

The logistic regression analysis indicated that the severity of UI, as measured by the ICIQ-SF, could be a significant predictor of functional balance, as evaluated by the BBS. This result suggests that interventions aimed at improving core musculature and reducing incontinence symptoms could positively impact the functional balance of patients with MS, although further studies are required to confirm this relationship (12, 44).

This study offers relevant findings but presents several limitations. First, although the sample size calculation indicated 29 participants, only 27 were analyzed, slightly reducing statistical power, especially for subgroup correlations. However, observed effect sizes suggest a modest impact on primary associations. Second, the cross-sectional design precludes causal inference; longitudinal studies are needed for stronger evidence. Third, multiple correlations were explored without formal multiplicity correction due to the study’s exploratory nature and limited sample, so marginal *p*-values should be interpreted cautiously. Similarly, logistic regression results are limited by BBS dichotomization and small sample size, which restricted model stability and adjustment for confounders.

The cohort included different MS phenotypes, introducing heterogeneity. Analyses were not stratified to preserve power, larger cohorts are proposed to address this. Additionally, potential confounders (physical activity, BMI, disease duration, spasticity, medication) were not recorded or adjusted for, despite their known influence on fatigue, balance, and urinary symptoms. Larger, stratified cohorts and multivariable models are recommended to confirm and refine these preliminary associations.

In conclusion, these findings highlight the importance of core musculature, particularly the TA, in the relationship between UI, balance, and quality of life in patients with MS. Targeted interventions focusing on TA and pelvic floor strengthening could not only improve incontinence symptoms but also enhance postural stability and functionality in this population, offering a promising therapeutic approach.

## Data Availability

The raw data supporting the conclusions of this article will be made available by the authors, without undue reservation.
