# Supplementary material for: Urinary incontinence, core morphology and their impact on balance and fatigue in multiple sclerosis: an observational study
Source: Front Neurol. 2026 Jan 29;17:1708066. doi: 10.3389/fneur.2026.1708066 (PMC12893960; doi:10.3389/fneur.2026.1708066)
Supplement: Supplementary file 1 [file Image_1.pdf]

Supplementary Figures S1–S6

Note: All figures correspond to baseline assessments (pre-intervention).

Supplementary Figure S1. Baseline MFIS (Fatigue) in participants with and without urinary incontinence.

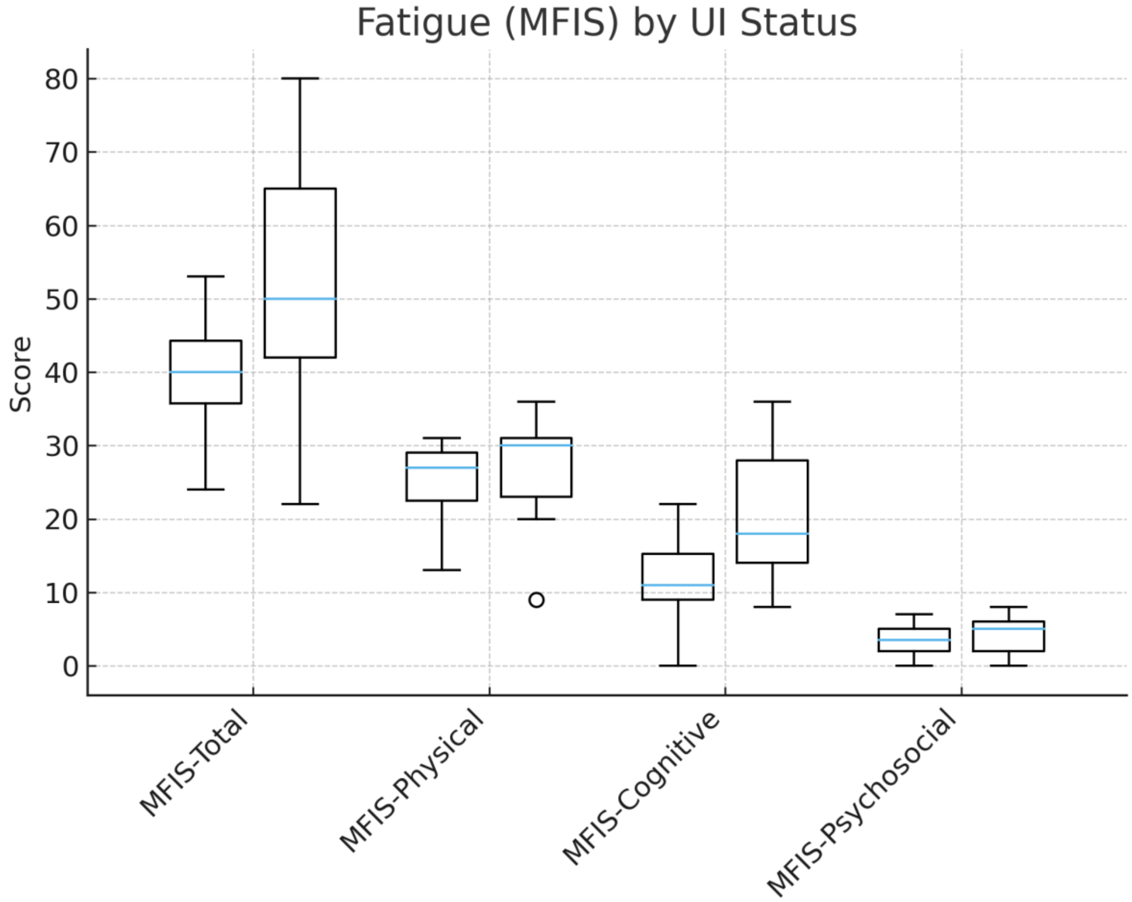

MFIS: Modified Fatigue Impact Scale; UI: urinary incontinence.

Supplementary Figure S2. Baseline balance (TIS and BBS) in participants with and without urinary incontinence.

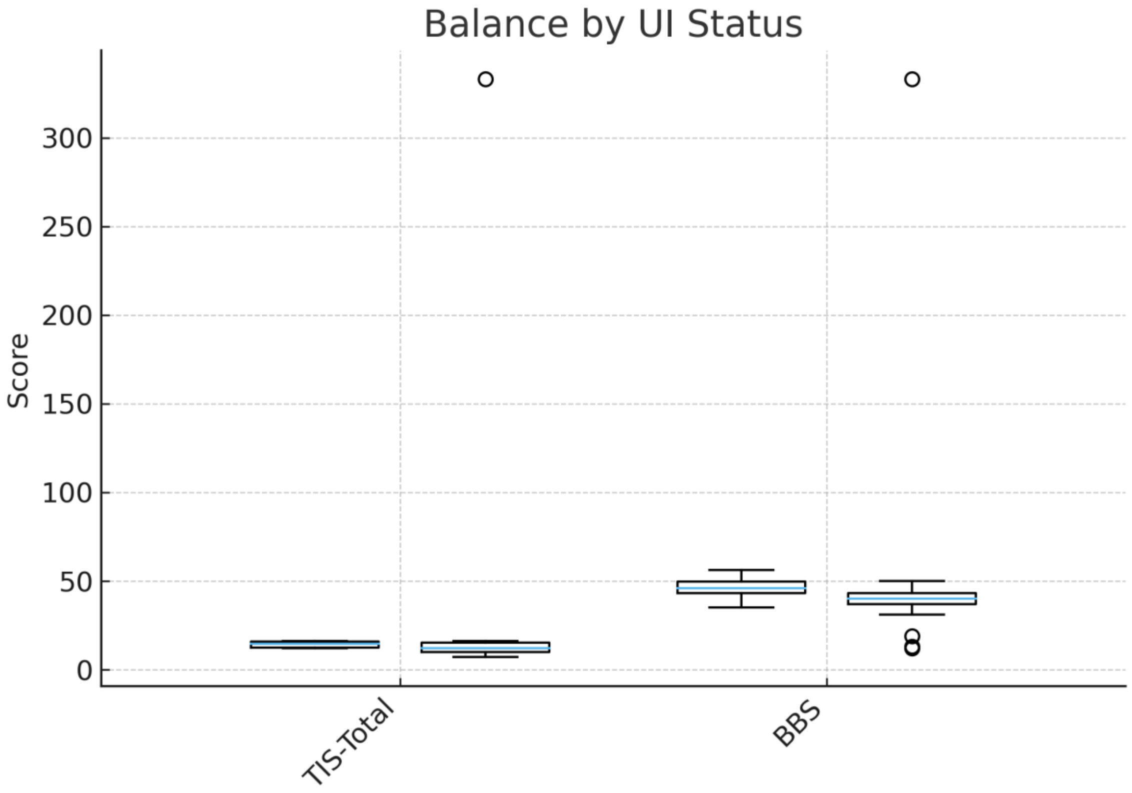

TIS: Trunk Impairment Scale; BBS: Berg Balance Scale, UI: urinary incontinence.

Supplementary Figure S3. Baseline abdominal muscle thickness (TA, IO, EO) in participants with and without urinary incontinence.

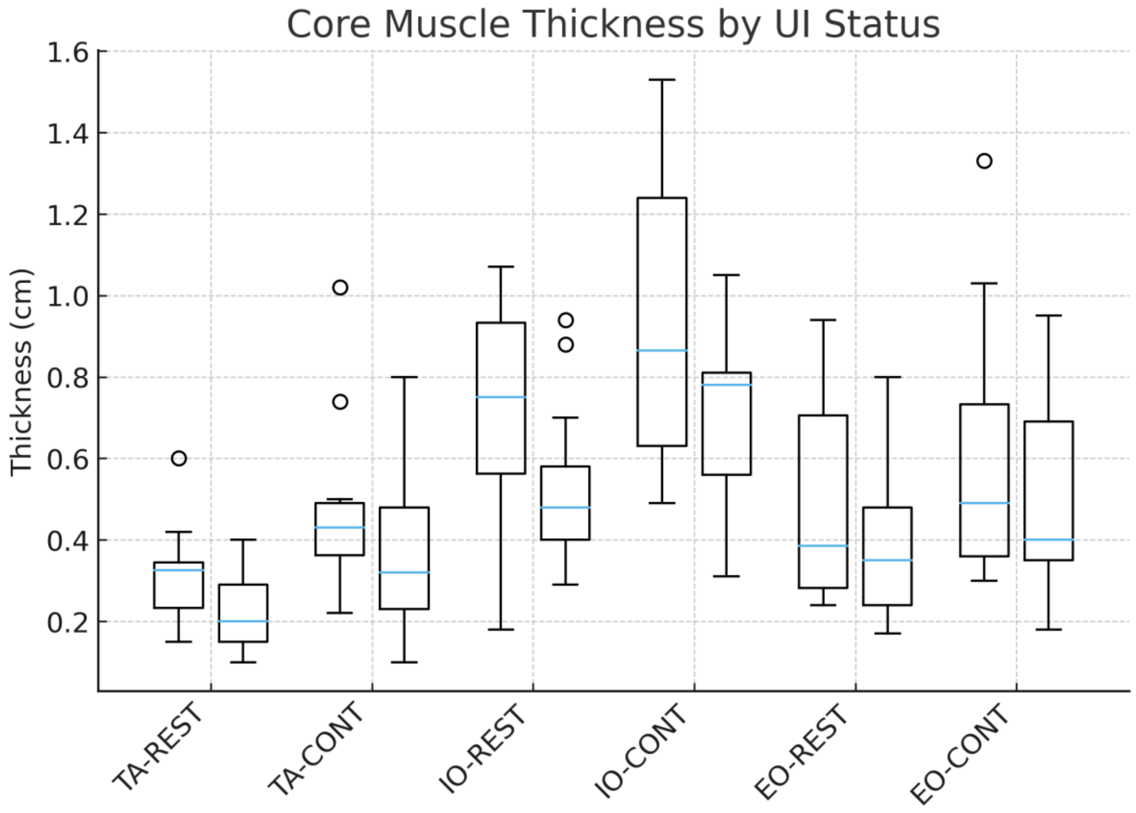

TA: transversus abdominis; IO: internal oblique; EO: external oblique.

Supplementary Figure S4. Baseline correlation between ICIQ-SF and BBS.

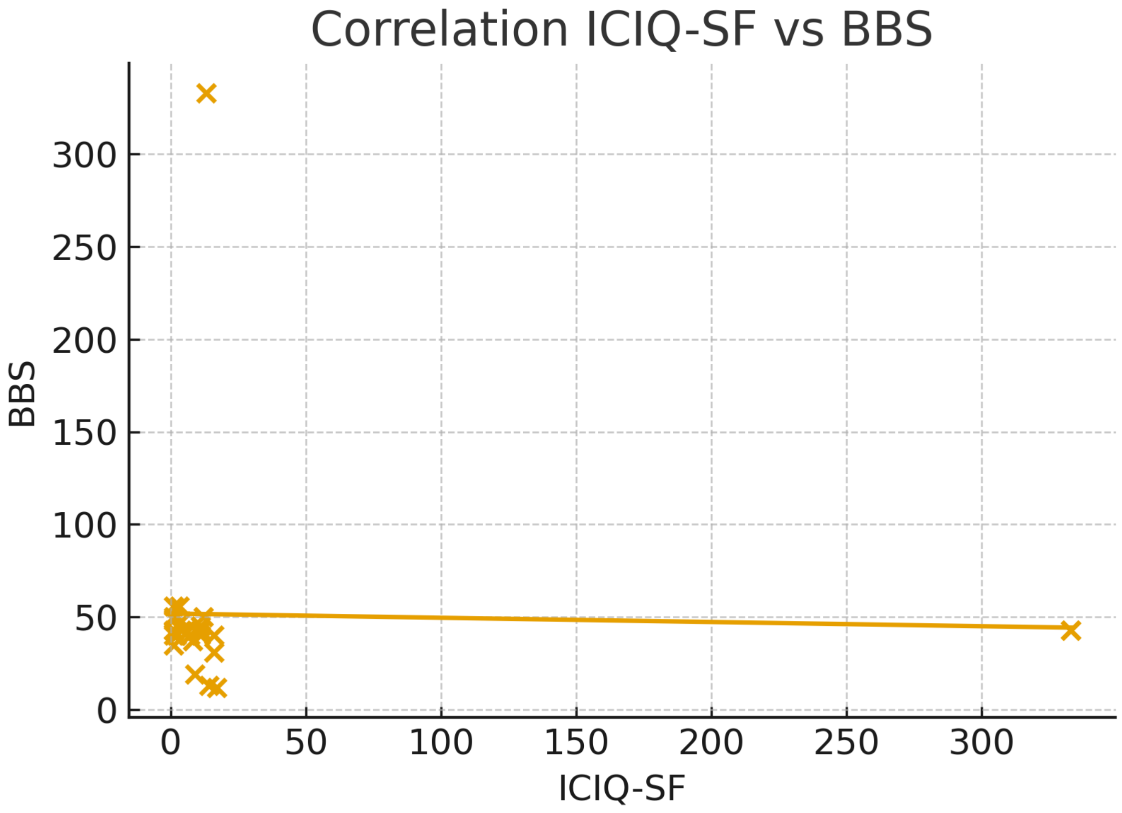

ICIQ-SF: International Consultation on Incontinence Questionnaire – Short Form; BBS: Berg Balance Scale

Supplementary Figure S5. Baseline correlation between ICIQ-SF and TIS-Total.

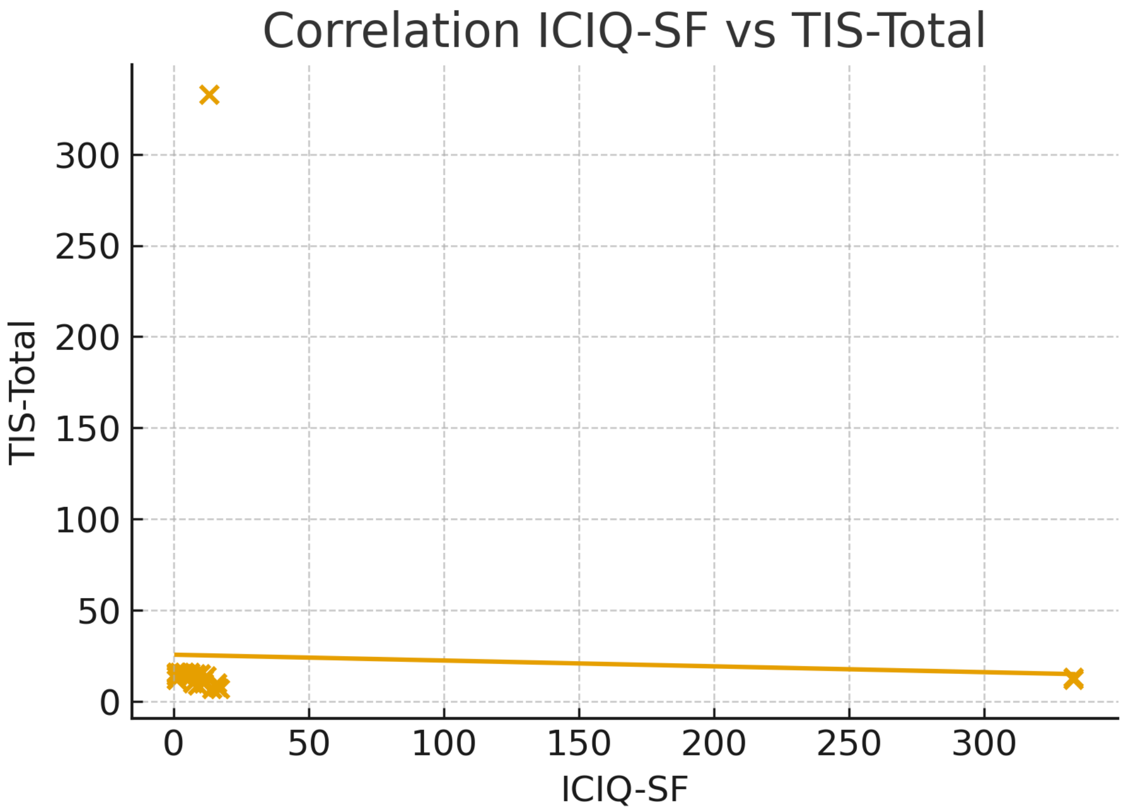

ICIQ-SF: International Consultation on Incontinence Questionnaire – Short Form; TIS-Total: Trunk Impairment Scale-Total

Supplementary Figure S6. Baseline correlation between ICIQ-SF and MFIS-Total.

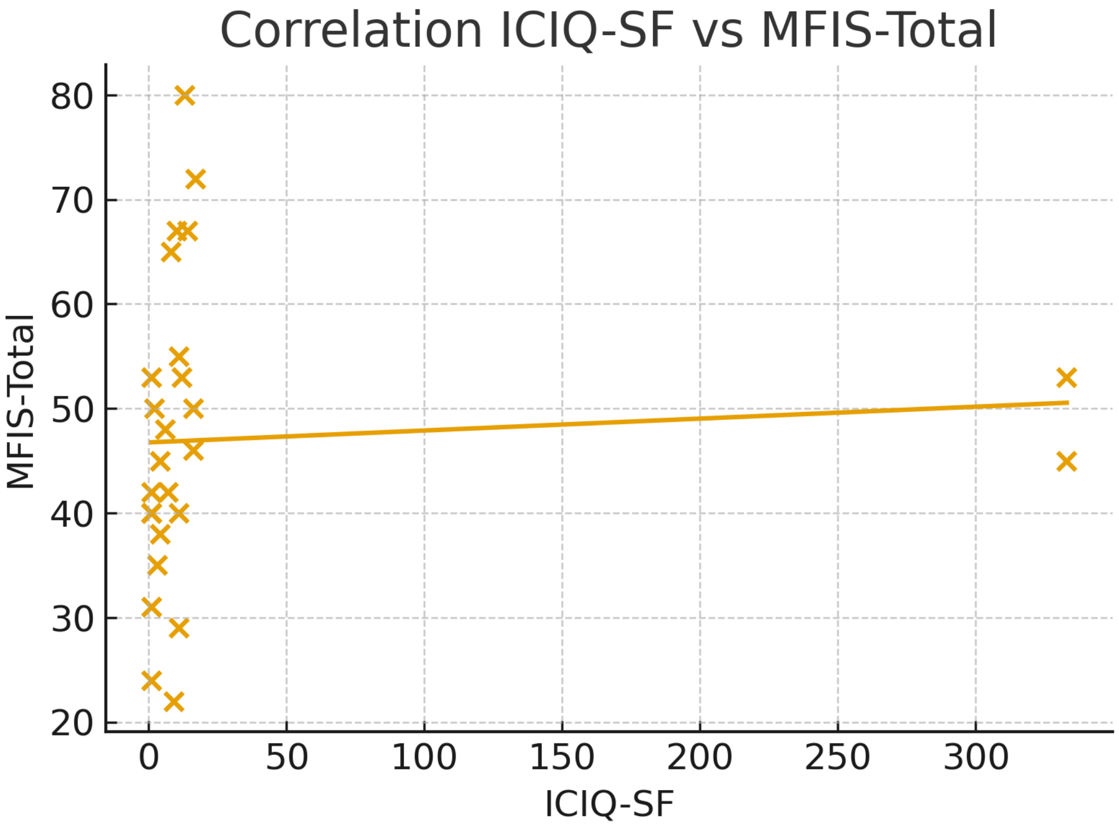

ICIQ-SF: International Consultation on Incontinence Questionnaire – Short Form; MFIS-Total: MFIS: Modified Fatigue Impact Scale-Total
